# Novel Molecular Targets in Malignant Diseases of Digestive System

**DOI:** 10.1155/2013/568280

**Published:** 2013-12-22

**Authors:** Chunping Jiang, Youmin Wu, Qiang Xia, Qin Huang

**Affiliations:** ^1^Department of Hepatobiliary Surgery, Affiliated Drum Tower Hospital, School of Medicine, Nanjing University, Nanjing, Jiangsu 210008, China; ^2^Intra-Abdominal Transplant and Hepatobiliary Surgery, Westchester Medical Center of New York Medical College, Valhalla, NY 10595, USA; ^3^Department of Transplantation and Hepatic Surgery, Renji Hospital, School of Medicine, Shanghai Jiaotong University, Shanghai 200127, China; ^4^Harvard Medical School, Boston, MA 02132, USA

Digestive malignancies remain one of the leading causes of cancer-related death worldwide despite the fact that increasing clinical and biological knowledge has emerged. Among all the newly discovered cancer cases, colorectal cancer, stomach cancer, liver cancer, and esophagus cancer rank in the front and the mortality rate for them also tops the list. The poor prognosis of digestive tumors is partially due to late diagnosis, delayed initiation of treatment, and unsatisfactory reaction to cancer therapies. Many molecular markers have been discovered for early diagnosis and better therapeutic outcomes from which we do benefit a lot. However, diagnosis and treatment of alimentary cancers require further optimization. We believe further study of novel molecular targets for early detection and better treatment would be helpful to reduce mortality rate and to improve the prognosis of malignant diseases of digestive system.

In this current issue, we focus on recent advances in the research of novel molecular targets which would help reveal the possible mechanism of tumorigenesis, progression, metastasis, and recurrence of digestive malignancies. The potential value of these molecular targets in cancer therapy is also highlighted. We present 10 articles on novel molecular targets in digestive system of which six articles discuss the molecular markers in hepatocellular carcinoma, three articles discuss the molecular makers in gastric carcinoma, and one makes a comprehensive review on one anticancer target in digestive system cancer therapy.

In the paper entitled “H-Ras oncogene expression and angiogenesis in experimental liver cirrhosis,” by G. Ö. Elpek et al. evaluated the relation between H-Ras expression and angiogenesis in liver cirrhosis which can progress to liver carcinoma. The oncogene H-Ras is elevated in liver cirrhosis and correlates significantly with angiogenesis.

“LEPREL1 expression in human hepatocellular carcinoma and its suppressor role on cell proliferation” by J. Wang et al. found that LEPREL1 was downregulated in hepatocellular carcinoma (HCC) tissues both in mRNA and protein levels, and the down-regulation was not associated with conventional clinical parameters of HCC. LEPREL1 could serve as a potential tumor suppressor gene by inhibiting HCC cell proliferation.

The research paper “Expression of potential cancer stem cell marker ABCG2 is associated with malignant behaviors of hepatocellular carcinoma” by G. Zhang et al. found that ABCG2 could probably function as a liver cancer stem cell marker because of its close relationship with tumorigenesis and also because it could promote cell proliferation, drug resistance, and metastasis. This molecule may represent an attractive target for the innovation of cancer stem cell-directed therapy for HCC.

In “Study of RNA interference targeting NET-1 combination with sorafenib for hepatocellular carcinoma therapy *in vitro* and *in vivo*,” S. He et al. found that the interference of NET-1 could enhance the anticancer effect of sorafenib. The interference of NET-1 could lead to impaired ability of proliferation and migration and could induce apoptosis in HCC cell line. NET-1 may be a promising molecular target to develop adjuvant therapy in combination with the only effective targeted drug, sorafenib for HCC.

Another two articles talked about the function of microRNA in HCC. X.-Y. Huang et al. showed that miR-29 was upregulated in HCC and correlated with poor outcomes of HCC. It might function by promoting cell proliferation and inhibiting cell apoptosis. The work by Z. Wang et al. clarified the association of miR-499 and miR-34b/c polymorphisms with susceptibility to HCC and got to the final conclusion that rs3746444 was not associated with susceptibility to HCC while rs4938723 was associated with increased HCC risk.

“Mast cells positive to tryptase and c-kit receptor expressing cells correlates with angiogenesis in gastric cancer patients surgically treated” by M. Ammendola et al. studied the angiogenesis in gastric cancer and found that MCTP and c-kitR-EC correlated positively with microvascular density. Drugs against c-kitR and tryptase could be promising agents in antiangiogenic therapy.

“Significance of glutathione peroxidase 1 and caudal-related homeodomain transcription factor in human gastric adenocarcinoma,” J. J. Han et al. demonstrated GPX1 and CDX2 might participate in the carcinogenesis, differentiation, and progression of gastric adenocarcinoma, and CDX2 might be an independent prognostic factor.

“Indirect comparison showed survival benefit from adjuvant chemoradiotherapy in completely resected gastric cancer with D2 lymphadenectomy” by Q. Yang et al. confirmed the role of adjuvant chemoradiotherapy in D2-resected gastric cancer patients with discussion of underlying molecular mechanism of this benefit.

The review article “PP2A-mediated anticancer therapy” by W. Chen et al. made a general review of the tumor suppressor PP2A by focusing on PP2A structure and the possible mechanism of its participation in anticancer therapy.

In summary, this special issue presents several intriguing achievements in the field of novel molecular targets in digestive malignancies which we hope could be utilized in the future for early diagnosis and treatment.

